# Properties of Cadmium-(*bis*)dodecylthiolate and Polymeric Composites Based on It

**DOI:** 10.3390/ma8125487

**Published:** 2015-12-11

**Authors:** Nadezhda Agareva, Anton A. Smirnov, Andrey Afanasiev, Semen Sologubov, Alexey Markin, Evgenia Salomatina, Larisa Smirnova, Nikita Bityurin

**Affiliations:** 1 Institute of Applied Physics of Russian Academy of Sciences, 46 Ul'yanov Street, 603950 Nizhny Novgorod, Russia; agareva@ufp.appl.sci-nnov.ru (N.A.); antonsmirnov@ufp.appl.sci-nnov.ru (A.A.S.); ava@ufp.appl.sci-nnov.ru (A.A.); 2 Department of Chemistry, N.I. Lobachevsky State University of Nizhny Novgorod, 23 Gagarin Ave, Building 5, 603950 Nizhny Novgorod, Russia; s.slg90@gmail.com (S.S.); markin79@mail.ru (A.M.); ox-eye_daisy23@mail.ru (E.S.); smirnova_la@mail.ru (L.S.)

**Keywords:** composite materials, poly(methyl methacrylate), cadmium-(*bis*)dodecylthiolate, photoinduced CdS nanoparticles, photoluminescence, thermophysical properties

## Abstract

We study the thermo-physical and photoluminescence (PL) properties of cadmium-(*bis*)dodecylthiolate (Cd(C_12_H_25_S)_2_). Significant attention is drawn to characterization of Cd(C_12_H_25_S)_2_ by different methods. The laser-induced PLs of Cd(C_12_H_25_S)_2_ and Cd(C_12_H_25_S)_2_/(polymethyl methacrylate) (PMMA) composites are studied. Samples of Cd(C_12_H_25_S)_2_/PMMA are synthesized by the polymerization method. Ultraviolet (UV)-pulsed laser irradiation of the samples under relatively small fluences leads to the formation of induced PL with the maximum near the wavelength of 600 nm. This process can be attributed to the transformation of Cd(C_12_H_25_S)_2_ within the precursor grains. Another PL peak at 450–500 nm, which appears under the higher fluences, relies on the formation of CdS complexes with a significant impact of the polymer matrix.

## 1. Introduction

Nanocomposite materials containing metal or semiconductor particles are of primary interest for researchers because of the wide range of their applications [1,2]. *In situ* methods of semiconductor nanoparticle growth can be performed with the help of precursors admixed into polymer matrices. Ultraviolet (UV) lamp or laser irradiation of the materials consisting of a polymer matrix with precursors of different kinds, possibly followed by annealing, can result in the creation of nanoparticles within the irradiated domains. Such photoinduced nanocomposites are promising for photonics applications due to the strong alteration of their optical properties compared to the initial non-irradiated materials [1,3,4,5].

The precursors are molecules containing metal atoms [1,3,4,5] (in the case of plasmon media) or atoms constituting a semiconducting material [2,6] (in the case of excitonic media). The precursors, when exposed to radiation, decompose with the subsequent formation of either plasmon or semiconducting nanoparticles, respectively, in the solid or liquid media, for example in the polymer matrix. The photoinduced plasmon nanocomposites with polymer matrices have been studied for a decade [3,4,5]. Recently, the creation and study of exciton-photoinduced nanocomposites have come to the forefront [7,8,9,10,11,12,13,14,15,16,17,18].

The essential point in these studies is the proper choice of the precursor species. The most popular precursors for laser-induced CdS nanoparticle formation are cadmium-(*bis*)thiolates. CdS particles can be prepared from them through thermal as well as laser decomposition. Thermal treatment of cadmium-(*bis*)thiolates is described in [8,9]. Laser processing is a more promising way to form particles in polymer matrices. It provides the ability to irradiate the chosen area, which is of interest for laser writing [10], and includes a great number of variable parameters, such as pulse repetition rate, wavelength, and laser fluence, for the processing optimization. The process of laser-induced semiconductor nanoparticle formation from cadmium-(*bis*)thiolates is described in [11,12,13,14]. The elucidation of the properties of the precursor is of fundamental importance. In this paper, we study the thermal properties of cadmium-(*bis*)dodecylthiolate (Cd(C_12_H_25_S)_2_) in detail. We also focus on the thermal and optical properties of the composites of polymethyl methacrylate (PMMA) containing Cd(C_12_H_25_S)_2_. (Cd(C_12_H_25_S)_2_/PMMA) prepared by radical polymerization as bulk samples. Most of the previous studies deal with film samples, whereas bulk samples allow for three-dimensional (3D) laser structuring applications [19]. We also investigated photoluminescence (PL) spectra of a thermally treated and laser-irradiated precursor powder and compare them with similar properties of the laser-irradiated (Cd(C_12_H_25_S)_2_/PMMA) bulk samples.

## 2. Experimental Section

### 2.1. The Synthesis of Cadmium-(bis)dodecylthiolate

We used the following materials: Cadmium nitrate tetrahydrate Cd(NO_3_)_2_·4H_2_O, absolute ethanol, dodecanethiol, acetone, methyl methacrylate (MMA), and azo-*(bis)*isobutyronitrile (AIBN).

The synthesis of Cd(C_12_H_25_S)_2_ was performed in accordance with the modified technique described in [8,9,20]. Cd(NO_3_)_2_·4H_2_O and dodecanethiol were dissolved separately in absolute ethanol with concentrations 0.8 and 1.6 mol/L respectively. A dodecanethiol solution was added to an equal volume of the Cd(NO_3_)_2_·4H_2_O solution. The addition of the first drops of the dodecanethiol solution leads to the appearance of a white precipitate. The mixture was stirred intensively for 1 h. The precipitate was separated by decantation, rinsed two times in ethanol and centrifuged, then washed two times in acetone, centrifuged again, and dried to constant weight. The volume of the solvents used for each step of the precipitate product rinsing was equal to the total amount of two initial solvents. All steps were carried out at 25 °C in the air. The dried product was a white powder insoluble both in polar and non-polar organic solvents at 20–25 °C, but it is soluble in hot toluene at temperatures of up to 70 °C.

### 2.2. CHNS-Analysis of Cadmium-(bis)dodecylthiolate with General Formula Cd(C_12_H_25_S)_2_ and Molecular Weight of 514 Da

We performed elemental analysis of the cadmium compound synthesized with a Vario El cube CHNS elemental analyzer (Elementar Analysensysteme, Hanau, Germany) using the equipment of the Collective Usage Center “New Materials and Resource-Saving Technologies” (N.I. Lobachevsky State University of Nizhny Novgorod, project RFMEFI59414X0005). The weighing was performed with an A&D GR-202 analytical balance (A&D Company, Limited, Tokyo, Japan). Sulfanilamide used as the reference for the elemental analysis was standardized by the device manufacturer. A weighed portion of a sample to be analyzed ((1–1.5) ± 0.01 mg) was packed into a tin capsule and then placed in the automated sampler of the device. The subsequent combustion of the sample and absorption of the released CO_2_ were performed in automated mode. The analytical data were processed and the mass content of carbon in the sample was calculated by using original software provided by the device manufacturer. For each set of analyses, calibration was performed against a standard sulfanilamide sample.

Metrological characteristics [21] of the original Elementar’s procedure for determination of carbon in organic and inorganic samples were preliminarily verified. Sulfanilamide standardized by the manufacturer of the elemental analyzer and shipped with the device was used as the standard sample. It was shown that the average confidence interval δ estimated from 10 successive measurements of a 1–2 g weighed portion of sulfanilamide is 0.7 wt % (see Table 1). The systematic error estimated using the *t*-criterion is statistically nonsignificant. Metrological characteristics of the original Elementar’s procedure were used for determining the mass content of carbon in organic and inorganic samples (*n* = 10 and *P* = 0.95, with sulfanilamide as the reference).

**Table 1:** Metrological characteristics of the Elementar’s procedure.

Characteristics	Value
Found content, wt %	41.1
True content, wt %	41.8
*s*, wt %	1
±δ, wt %	0.7
*t*_p_, *f* = *n* − 1	2.3
*t*_found_	2.2

Weighing inaccuracy gave the main contribution to the random determination error. The relative root-mean-square (rms) deviation of balance readings in the primary verification for a weighed portion mass of 1.5 mg is 1.5%. This value constitutes about two-thirds of the relative rms deviation of the result of single determination of the mass fraction of carbon in a sample (2.4%).

The results of the cadmium compound analysis are presented in Table 2. Thus, the product of the synthesis is Cd(C_12_H_25_S)_2_. The purity of the compound was 99.9%.

**Table 2:** Elemental composition of Cd(SR)_2_.

Element	Theoretically Calculated Content of Elements in the Cadmium *bis*-(dodecylthiolate), wt %	Experimental Results of Elements Content in the Obtained Cd(SR)_2_, wt %
С	56.03	55.9 ± 0.40
H	9.73	10.0 ± 0.40
S	12.45	12.49 ± 0.17
Cd	21.78	21.61 ± 0.40

### 2.3. Differential Scannig Calorymetry (DSC) and Thermogravimetric Analysis (TGA) 

To study the thermal behavior of Cd(C_12_H_25_S)_2_ and Cd(C_12_H_25_S)_2_/PMMA samples, we used a differential scanning calorimeter (model: DSC 204 F1 Phoenix, Netzsch Gerätebau, Selb, Germany). The calorimeter was calibrated and tested against melting of *n*-heptane, mercury, tin, lead, bismuth, and zinc. Standard uncertainty for temperature was *u*(*T*) = 0.5 °C and the relative standard uncertainty for the enthalpies of transformations was *u_r_*(Δ*_fus_H_m_*) = 0.01. The temperatures and the enthalpies of transitions were evaluated according to the standard Netzsch Software Proteus procedure [22,23]. The measurement was carried out in the argon atmosphere.

The thermogravimetric (TG) analysis of Cd(C_12_H_25_S)_2_ was done using a thermal microbalance (model TG 209 F1 Iris, Netzsch Gerätebau, Germany). Thermogravimetric Analysis (TGA) was carried out in the range from 20 to 160 °C in the argon atmosphere. The thermal microbalance TG 209 F1 Iris allows fixing the mass change in ±0.1 μg. The average heating rate was 10 °C·min^−1^. The measuring technique of TGA was standard, according to Netzsch Software Proteus (Netzsch Gerätebau, Selb, Germany).

### 2.4. Synthesis of the Bulk Polymeric Samples Containing Cadmium-bis(dodecylthiolate)

Bulk radical polymerization of MMA containing 0.02 M of AIBN and 1% to 3% (weight) of Cd(C_12_H_25_S)_2_ was carried out at 70 °C. Cd(C_12_H_25_S)_2_ is insoluble in MMA. To achieve a uniform mixture of this compound and monomer, and to avoid its precipitation during the synthesis of polymethyl methacrylate (PMMA), a glass ampoule filled with reaction mixture was sonicated for 1 h at room temperature without the radical initiator. Then the reaction mixture was heated to 70 °C and AIBN was introduced. Sonication was continued up to the onset of the gel effect and was stopped when the reaction mixture became viscous enough to avoid the precipitation of Cd(C_12_H_25_S)_2_. The polymerization was proceeded for 4 h. Then the reaction mixture was heated up to 140 °C and kept at that temperature for 1 h.

The molecular weight distributions were obtained by gel permeation chromatography (Prominence LC-20VP, Shimadzu Corporation, Tokyo, Japan): the average molecular weight *M_w_* = 1.4·× 10^6^ g/mol, the polydispersity index was *M_w_*/*M_n_* = 4.15.

### 2.5. Laser Processing of Samples and Study of Their Luminescent Properties

The irradiation is performed with a Nd:YAG laser LS-2137 («Lotis ТII», Minsk, Belarus) operated at the fourth harmonic at a wavelength of 266 nm. The repetition rate is 1 laser pulse per second, and the laser fluence is chosen in the range 150–300 mJ/cm^2^. The diameter of the laser beam is about 1.5 mm. The samples are mounted on an optical table with three-coordinate adjustment for irradiation by the laser beam.

Luminescent properties of the obtained samples are tested by excitation with a laser diode at a wavelength of 405 nm. Scattered and luminescent light is collected with a condenser lens into the Ocean Optics QE65Pro spectrometer fiber probe (Ocean Optics, Dunedin, FL, USA). A sharp-edged low-pass spectral filter ET-425LP (Chroma Technology Corp, Bellows Falls, VT, USA) is used for the excitation wavelength cutoff. The spectrum was analyzed using a spectrometer calibrated with an HL2000 lamp (Ocean Optics). Thermal power sensor Thorlabs S302C (Thorlabs GmbH, Dachau, Germany) is used for the optical power and energy detection. Confocal microscope Zeiss LSM710 (Carl Zeiss Microscopy GmbH, Jena, Germany) is used for obtaining microimages of the samples with irradiated area and for detection of the local luminescent spectral characteristics.

## 3. Results and Discussion

### 3.1. Thermophysical Characteristics of Cadmium-bis(dodecylthiolate)

Different metal thiolates are widely used precursors for obtaining the corresponding metal sulfides in various polymer matrices. The purity of the compounds being studied is of considerable importance. Therefore, it is necessary to have a rapid method of purity control. If reliable reference data are available, the simplest way to identify the purity of a solid-state material is determination of the melting point by the capillary method. Melting points of a wide range of metal thiolates are reported in [20].

**Figure 1:**
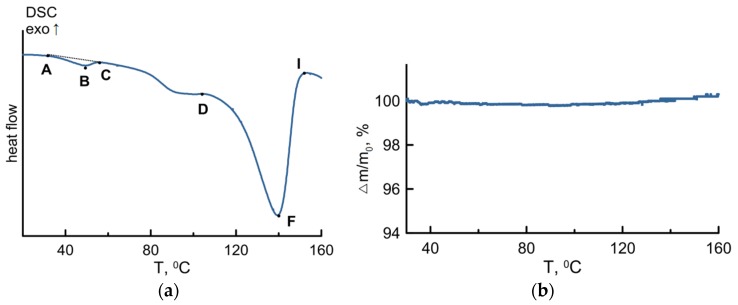
Differential scanning calorimetry (DSC) (**a**) and thermogravimetric (TG) (**b**) measurements of Cd(C_12_H_25_S)_2_ powder in an argon atmosphere.

However, we did not observe the formation of liquid droplets in the glass capillary filled with Cd(C_12_H_25_S)_2_ at 42 °C as reported in [20]. It can be assumed that the authors of [20] did not make a convincing conclusion about the nature of the first transition on the curve obtained by differential scanning calorimetry (DSC) of metal thiolates. We consider the DSC data analysis of Cd(C_12_H_25_S)_2_ (Figure 1a) in more detail.

The thermal stability of Cd(C_12_H_25_S)_2_ was studied by the TG method (Figure 1b). According to these results, the detectable weight loss in the sample is obtained at temperatures above 160 °C. The DSC data give evidence for two endothermic effects in different temperature ranges from 36 to 56 °С and from 112 to 152 °С, which are reproduced in the iterated stages of the cooling-heating of Cd(C_12_H_25_S)_2_ from 20 to 150 °C. Both effects can be attributed to the phase transition. The transition enthalpies amount to Δ*H*°_1_ = 2.23 mJ/mol and Δ*H*°_2_ = 0.12 J/mol. The enthalpy of the first transition corresponds to the enthalpy of conformal transitions in the Cd(C_12_H_25_S)_2_ molecule in solid state [24]. The second transition relies on the melting of the studied compound. Taken together, the DSC and TG data point to the incongruent melting of Cd(C_12_H_25_S)_2_, which is in contradiction to the data available in the literature [20].

It should be noted that when analyzing the DSC results, it is necessary to take into account that the endothermic processes may indicate not only the melting, but also other physical processes, and conformal transitions in particular. The numerical values of the corresponding enthalpies should be taken into account.

**Figure 2:**
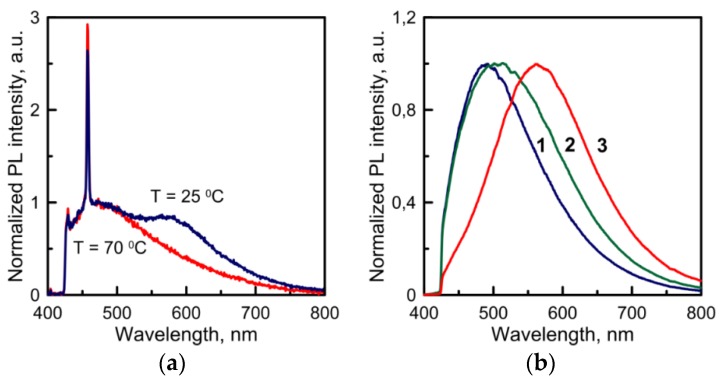
(**a**) Normalized PL spectra of Cd(C_12_H_25_S)_2_ powder measured at 25 and 70 °C. (**b**) The evolution of normalized PL spectra of Cd(C_12_H_25_S)_2_ powder annealed at 240 °C: 1–5 min, 2–40 min, 3–60 min, measured at room temperature.

For the purpose of studying the thermal effect on the luminescent properties of Cd(C_12_H_25_S)_2_, we study the evolution of its PL spectra after heating in the furnace. This effect is reversible when the temperature is less than the melting point. The spectra of a “hot” sample and of the same sample at room temperature have different forms (see Figure 2a). At room temperature, two wide maxima can be observed: one at 460 nm and another at 580 nm. The second maximum disappears in the case of high temperature, but it comes back as the sample is cooled. We suppose this effect can be attributed to the conformal transition in the range 36–56 °C.

When the sample is heated to the melting point (about 140 °C), the maximum at 580 nm is no longer obtained even after the cooling. If the sample is heated at higher temperatures, the melting process will become more intense, with irreversible transformation of the PL spectrum. The change of color from white to yellow and orange can be observed. At high temperatures, the sample is liquid and transparent, and it becomes opaque when cooled. We keep the sample at 240 °C for 1 h. The photoluminescence intensity increases dramatically compared to the initial spectrum. The maximum of the PL signal moves towards the higher wavelengths, from 500 to 570 nm, in the course of thermal annealing (Figure 2b).

### 3.2. Luminescence Properties of the Laser-Irradiated Cadmium-bis(dodecylthiolate)

The main feature of cadmium-(*bis*)thiolates is their poor solubility. That is why we have the precursor dispersed in the matrix with grains of the average size of a few microns.

Therefore, it is useful to compare the effect of laser radiation on the composite PMMA/Cd(C_12_H_25_S)_2_ samples and the results of irradiation of a pure precursor. If the effect of laser radiation on the polymer samples is reduced to the modification of the precursor within the grains, then the luminescent properties of the laser-irradiated pure Cd(C_12_H_25_S)_2_ powder and polymer PMMA/Cd(C_12_H_25_S)_2_ samples will be close to each other.

**Figure 3:**
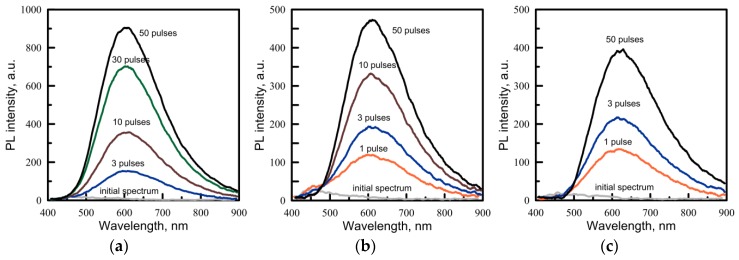
The evolution of PL emission spectra of Cd(C_12_H_25_S)_2_ powder irradiated with various numbers of UV laser pulses of 266 nm wavelength. The laser fluence is 150 mJ/cm^2^ (**a**), 190 mJ/cm^2^ (**b**), 290 mJ/cm^2^ (**c**).

Two types of samples are chosen: pure Cd(C_12_H_25_S)_2_ powder and bulk PMMA samples with 1% (weight) of Cd(C_12_H_25_S)_2_ (fine polished discs 10 mm in diameter and about 1 mm thick). For the irradiation experiment, we used laser pulses with fluences 150, 190, 290 mJ/cm^2^ to compare their effects on the precursor and the PMMA/Cd(C_12_H_25_S)_2_ samples. The initial PL signals of obtained samples are weak (see Figure 3). 

If a pure precursor powder is irradiated, then the induced PL can be observed even after the first pulse (Figure 3). When irradiated with many pulses, the signal increases and tends toward saturation. The maximum of the PL spectrum is observed at 600–615 nm, and this wavelength depends neither on the laser fluence nor on the number of pulses. This spectrum is close to that obtained by the thermal annealing described above. This implies that the laser heating of the precursor could play an important role in the material alteration by the laser treatment. During the laser processing, we observe that white powder changes its color to yellow. A brief summary of features of the PL spectra of Cd(C_12_H_25_S)_2_ subjected to heating and laser irradiation is given in Table 3.

**Table 3:** Features of the photoluminescence (PL) spectra related to the Cd(C_12_H_25_S)_2_ powder treatment.

Way of Treatment	Description of PL Spectrum
Heating at 70 °C	Reversible transformation
Heating at 240 °C	Irreversible increase in PL signal with the maximum movement towards the longer wavelengths from 500 to 570 nm
Laser irradiation at 266 nm	Increase in PL signal with the maximum wavelength at 600–615 nm

### 3.3. DSC and TG Characterizations of the Cd(C_12_H_25_S)_2_/PMMA Samples

**Figure 4:**
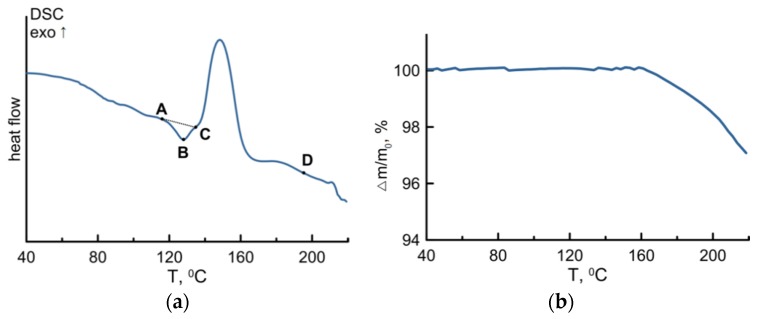
DSC (**a**) and TG (**b**) measurements of the Cd(C_12_H_25_S)_2_/PMMA sample in an argon atmosphere.

DSC and TG measurements were performed for the Cd(C_12_H_25_S)_2_/PMMA sample before the laser irradiation. DSC measurements revealed an endothermic effect in the temperature range of 117–136 °C with a transition temperature of 128 °C and enthalpy of 1.61 J/g (see Figure 4a, region ABC). A similar endothermic effect in the range of 106–153 °C for a pure Cd(C_12_H_25_S)_2_ powder was discussed above. This effect was attributed to the incongruent melting of the precursor. In the case of a Cd(C_12_H_25_S)_2_/PMMA sample, the transition temperature is lower, which is obvious if the interaction between Cd(C_12_H_25_S)_2_ and the PMMA matrix is taken into account. Thermal destruction of the sample takes place at a temperature of about 200 °C (point D), which can be seen from the TG curve (Figure 4b). The exothermic effect in the temperature range of 135–165 °C is peculiar for the sample of PMMA with a precursor and is observed neither for the pure PMMA sample, nor for the Cd(C_12_H_25_S)_2_ powder. It can be due to the chemical interaction between the products of thermal degradation of Cd(C_12_H_25_S)_2_ and the polymer matrix. However, this needs special analysis.

### 3.4. Luminescence Properties of the Laser-Irradiated Cd(C_12_H_25_S)_2_/PMMA Samples

**Figure 5:**
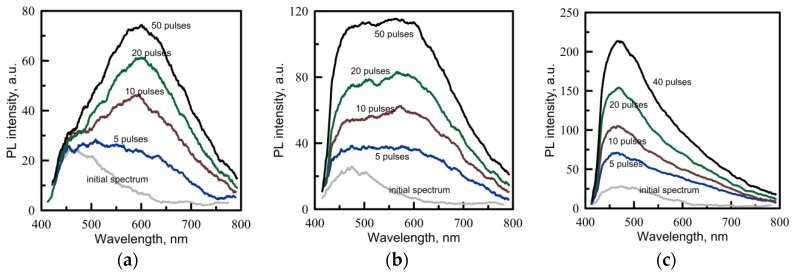
The evolution of photoluminescence (PL) emission spectra of 1% Cd(C_12_H_25_S)_2_/PMMA samples irradiated with various numbers of UV laser pulses. The laser fluence is 150 mJ/cm^2^ (**a**), 190 mJ/cm^2^ (**b**), and 290 mJ/cm^2^ (**c**).

The irradiation of Cd(C_12_H_25_S)_2_/PMMA samples with a fluence of 150 mJ/cm^2^ leads to the induced PL signal at 600 nm, which probably corresponds to the modification of a pure precursor within the grains (Figure 5a). If the laser fluence is higher, then the induced PL spectra differ from the spectra of the irradiated pure precursor. We observe an increase in PL both at 475–500 nm and near 600 nm (Figure 5b,c). When the fluence is 290 mJ/cm^2^, the main increase in the PL signal occurs near 500 nm, while the peak is saturated at 600 nm.

Thus, when the pulses are weak, the spectrum of induced PL relies on the transformation of a pure precursor. At the higher fluences, the impact of the matrix is significant. The band gap of pure CdS corresponds to a wavelength of about 515 nm. Therefore, the formation of CdS particles is usually revealed through the PL spectrum located at a wavelength shorter than 500–520 nm. The peaks at the longer wavelengths rely on the trap states, whose nature depends on the particle environment and defects on their surface [25,26]. In our work, we find that, in a pure irradiated precursor, there is no PL peak near 500 nm, but the luminescence at 600 nm is very intense. It should be noted that the luminescence in this range (located near 600 nm) has been observed in [19,26]. The authors of [26] connect this emission with the surface Cd having sulfur vacancy sites. In our case, it could be connected with the defect states, not necessarily at the surface but also within the bulk of the created clusters. In the Cd(C_12_H_25_S)_2_/PMMA samples, the PL peak typical of CdS is observed only for fairly strong irradiating pulses.

To explain the observed phenomena, we assume that the major part of the precursor distributed in the PMMA matrix constitutes agglomerates because of its poor solubility in this polymer, while the other part is better dispersed within the matrix. These components may have different effects on the PL properties of the sample under laser irradiation.

The sufficiently large agglomerates could be considered as inert filling compounds which have the properties of pure Cd(C_12_H_25_S)_2_ and give a PL signal with the maximum at 600–620 nm. Laser irradiation of the relatively small grains of Cd(C_12_H_25_S)_2_ with a high value of the relative surface area and strong intermolecular interaction with the PMMA matrix results in the appearance of a PL band at about 500 nm. The latter band can be associated with the CdS nanoparticles having a smaller content of trapped states.

**Figure 6:**
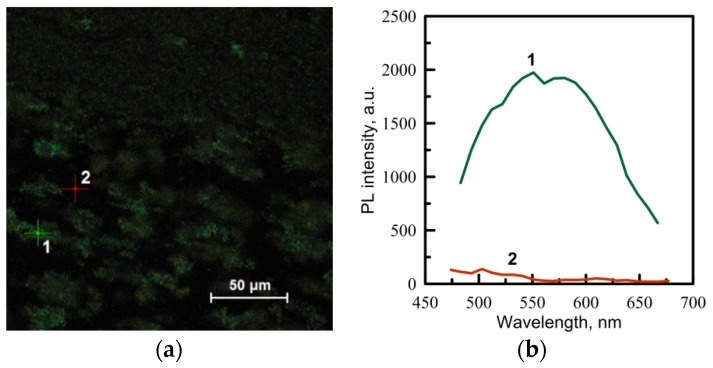
(**a**) Fluorescence images of the irradiated area of a 1% Cd(C_12_H_25_S)_2_/PMMA sample. (**b**) PL spectra from two different points, 1 and 2, of the region exposed to laser irradiation.

Fluorescence images of the irradiated regions on the Cd(C_12_H_25_S)_2_/PMMA samples are presented in Figure 6a. One can see that the signal of luminescence is recorded from areas whose size is about a few microns. We associate them with the agglomerates of precursor grains of different sizes. Here, the precursor transformed into semiconductor clusters as a result of the laser irradiation. PL spectra form two different points of the irradiated area (Figure 6b). Point 1 is located within the precursor agglomerate, while point 2 belongs to the PMMA matrix. It should be noted that PMMA matrices without the precursor were also irradiated as control samples. The laser fluences that were used for the processing do not affect a clean matrix.

## 4. Conclusions

The melting points of different metal thiolates in the range of 40–42 °C given in [20] are doubtful. The corresponding DSC peak is attributed to conformal transitions in the hydrocarbon chain of metal thiolates.

Detailed experimental study of the thermal behavior of Cd(C_12_H_25_S)_2_ using two independent methods and their comparison with the previous data in the literature, as well as the numerical values of the energy of the first identified effect, conclusively rule out its interpretation as of the phase transition point, and the thermodynamic classification attributes it to conformal transitions in solid state. The results on the Cd(C_12_H_25_S)_2_ thermal behavior were confirmed by the studies of the luminescent properties of the Cd(C_12_H_25_S)_2_ powder carried out at several fixed values of temperature. Irradiation of pure Cd(C_12_H_25_S)_2_ by the UV laser at a wavelength of 266 nm (15 ns pulses) leads to the appearance of luminescence with the maximum at 600 nm, which is close to the result of thermal annealing of the precursor powder at temperatures higher than the incongruent melting point.

The polymeric glass samples containing Cd(C_12_H_25_S)_2_ have been obtained by MMA bulk polymerization. The main feature of cadmium-(*bis*)thiolates is their poor solubility. The precursor is dispersed in the matrix with grains having an average size of a few microns. This was confirmed by luminescent microscopy of the laser-irradiated samples. Irradiation of the Cd(C_12_H_25_S)_2_/PMMA samples at relatively small fluences results in the appearance of a PL spectrum with the maximum wavelength close to the maximum of the spectrum of a pure Cd(C_12_H_25_S)_2_ powder. This effect is due to the precursor transformation within the grains. A more intense maximum that appeared near 500 nm in the bulk samples irradiated under higher fluences can be attributed to the formation of CdS particles with a smaller content of trapped states. The difference in PL spectra of the pure precursor and bulk Cd(C_12_H_25_S)_2_/PMMA samples proves a significant role of the matrix in the process of the photoinduced composite formation.
